# New insights into extracellular and intracellular redox status in COVID-19 patients

**DOI:** 10.1016/j.redox.2022.102563

**Published:** 2022-12-02

**Authors:** Mohammad Javad Tavassolifar, Hamid Asadzadeh Aghdaei, Omid Sadatpour, Samaneh Maleknia, Sara Fayazzadeh, Seyed Reza Mohebbi, Fatemeh Montazer, Amirhassan Rabbani, Mohammad Reza Zali, Maryam Izad, Anna Meyfour

**Affiliations:** a Basic and Molecular Epidemiology of Gastrointestinal Disorders Research Center, Research Institute for Gastroenterology and Liver Diseases, Shahid Beheshti University of Medical Sciences, Tehran, Iran; b Department of Immunology, School of Public Health, Tehran University of Medical Sciences, Tehran, Iran; c Bioinformatics and Computational Omics Lab (BioCOOL), Department of Biophysics, Faculty of Biological Sciences, Tarbiat Modares University, Tehran, Iran; d Gastroenterology and Liver Diseases Research Center, Research Institute for Gastroenterology and Liver Diseases, Shahid Beheshti University of Medical Sciences, Tehran, Iran; e Department of Pathology, Firoozabadi Hospital, School of Medicine, Iran University of Medical Sciences (IUMS), Tehran, Iran; f Department of Transplant & Hepatobiliary Surgery, Taleghani Hospital, Shahid Beheshti University of Medical Sciences, Tehran, Iran; g Immunology Department, School of Medicine, Tehran University of Medical Sciences, Tehran, Iran; h MS Research Center, Neuroscience Institute, Tehran University of Medical Sciences, Tehran, Iran

**Keywords:** SARS-COV-2, Redox system, Nasopharyngeal, PBMCs, Immune system, COVID-19

## Abstract

**Background:**

The imbalance of redox homeostasis induces hyper-inflammation in viral infections. In this study, we explored the redox system signature in response to SARS-COV-2 infection and examined the status of these extracellular and intracellular signatures in COVID-19 patients.

**Method:**

The multi-level network was constructed using multi-level data of oxidative stress-related biological processes, protein-protein interactions, transcription factors, and co-expression coefficients obtained from GSE164805, which included gene expression profiles of peripheral blood mononuclear cells (PBMCs) from COVID-19 patients and healthy controls. Top genes were designated based on the degree and closeness centralities. The expression of high-ranked genes was evaluated in PBMCs and nasopharyngeal (NP) samples of 30 COVID-19 patients and 30 healthy controls. The intracellular levels of GSH and ROS/O_2_^•^ − and extracellular oxidative stress markers were assayed in PBMCs and plasma samples by flow cytometry and ELISA. ELISA results were applied to construct a classification model using logistic regression to differentiate COVID-19 patients from healthy controls.

**Results:**

*CAT, NFE2L2, SOD1, SOD2* and *CYBB* were 5 top genes in the network analysis. The expression of these genes and intracellular levels of ROS/O_2_^•^ ^−^ were increased in PBMCs of COVID-19 patients while the GSH level decreased. The expression of high-ranked genes was lower in NP samples of COVID-19 patients compared to control group. The activity of extracellular enzymes CAT and SOD, and the total oxidant status (TOS) level were increased in plasma samples of COVID-19 patients. Also, the 2-marker panel of CAT and TOS and 3-marker panel showed the best performance.

**Conclusion:**

SARS-COV-2 disrupts the redox equilibrium in immune cells and the upper respiratory tract, leading to exacerbated inflammation and increased replication and entrance of SARS-COV-2 into host cells. Furthermore, utilizing markers of oxidative stress as a complementary validation to discriminate COVID-19 from healthy controls, seems promising.

## Highlights

• SARS-COV-2 alters intracellular/extracellular redox balance in host immune cells.
• Oxidative stress markers are overexpressed in PBMCs of COVID-19 patients.
• The antioxidant defense was inhibited in the upper respiratory tract.
• Oxidative stress markers were proposed to construct diagnostic panels.

## Graphical abstract

**Figure:**
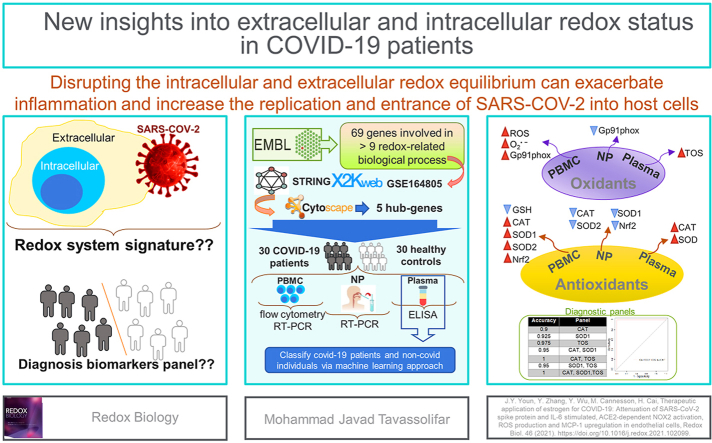

## 1 Introduction

Coronavirus disease 2019 (COVID-19) is a new respiratory illness caused by the ‘‘severe acute respiratory syndrome coronavirus 2’’ (SARS-CoV-2), with more than 490 million confirmed infections and more than 6 million deaths globally (April 2022). The majority of COVID-19 patients have mild to moderate symptoms, with 10% developing acute respiratory distress syndrome (ARDS), which is the primary cause of death in these individuals [1]. COVID-19 causes diffuse alveolar injury, chronic inflammatory infiltrates, and intra-alveolar fibrinous exudates, which are histopathologic alterations in the lungs [2]. Another characteristic of COVID-19 infection is triggering an uncontrolled cytokine response named “cytokine storm,” which is a hyper-inflammatory state of the disease [3]. Patients infected with SARS-CoV-2 have shown that releasing large amounts of pro-inflammatory cytokines, including IL-1β, IL-6, and TNF-α may promote free radical production and oxidative stress [4].

The redox homeostasis of a host cell is defined by the reactive oxygen species (ROS) and antioxidant molecules. The main types of ROS include superoxide anion (O_2_^•^^−^), hydroxyl radical (HO^•^), and hydrogen peroxide (H2O2), which are produced by nicotinamide adenine dinucleotide phosphate (NADPH) oxidases (NOXs) [5]. ROS are signaling molecules that cause the production of pro-inflammatory cytokines, and dysregulation of this response plays a crucial role in the progression of inflammation [6]. Furthermore, excessive reactive species generation is related to T and B cells activation and differentiation. Th1 cell development is induced by oxidative status, whereas the presence of reducing molecules stimulates Th2 responses [7]. In all tissues, cellular glutathione functions as a “master antioxidant”; the high concentration of the reduced form of cellular glutathione (GSH) is mainly involved in the regulation of various processes such as detoxification, antiviral defense, and immunological response [8,9]. Alterations in redox balance are described by the excess ROS production and failure of anti-oxidation defense systems, leading to oxidative stress, which is the main characteristic of several viral infections [10,11].

Several lines of evidence suggested a link between particular biological responses, such as inflammation and oxidative stress, and COVID-19 pathogenesis. Angiotensin-converting enzyme 2 (ACE2), as the receptor of SARS-CoV-2, needs a pro-oxidant environment to maintain its disulfide bonds intact, which is required for SARS-CoV-2 entrance into the host cells [12]. Also, IL-6 and S proteins induce oxidative stress via the activation of ROS generating enzymes, NOXs, causing a severe form of COVID-19 [13]. A further study demonstrated that infiltrating neutrophils, which are a characteristic of COVID-19 pathogenesis, can activate multiple pathways leading to increased cytokines and ROS formation [14]. Dysregulation of the host antioxidant response plays an important role in the pathogenesis of various viral diseases [15]. The antioxidant GSH can prevent cytokine storm in viral infection by inhibiting NF-kB and decreasing oxidant molecules [16]. Several antioxidant agents, including GSH, may reduce the interaction of host receptors with SARS-CoV-2 by regulating the cellular disulfide–thiol balance [17], suggesting a possible association between GSH pool depletion and an increased risk of severe COVID-19. Therefore, redox system might have a critical regulatory role in the proliferation/activation of immune cells as well as the virus entry into the host cells.

To highlight the upstream key drivers in redox systems and their interrelationships, it is imperative to take an integrative approach that combines multi-level biological data [18]. The biological processes (BPs), as the networks describing interactions between biomolecules, can provide the first level of biological information to understand the redox system via a top-down approach [19]. On the other hand, insight into the physical rewiring of protein-protein interactions (PPI) associated with redox system proteins can advance our knowledge to identify potential therapeutic targets [20]. Therefore, to investigate the influence of SARS-CoV-2 on the extracellular and intracellular redox states, we first constructed a network and identified key drivers of the redox system based on multi-level biological information, including 64 redox system-related BPs, PPI information, redox system-related transcription factors (TFs), and correlation coefficients. Correlation coefficients were obtained from published gene expression profiles of peripheral blood mononuclear cells (PBMCs) in COVID-19 patients and healthy individuals. The expression of the top ranked genes in the network was assessed in PBMCs and nasopharyngeal (NP) swab samples of COVID-19 patients. Furthermore, changes in intracellular and extracellular levels of metabolites and the activity of enzymes associated with redox status were explored in PBMCs and plasma samples of patients with COVID-19 compared to healthy controls. Ultimately, the classification was performed on ELISA results to discriminate cases against control samples, using logistic regression. A model was constructed for each combination of 1–3 markers and the models were evaluated using 5-fold cross-validation. The obtained accuracies were in the range of 0.9–1.

## 2 Materials and methods

### 2.1 Determination of potential key genes of the redox system in COVID-19 patients based on multi-level biological data: a bioinformatics approach

Multi-level biological information was applied to define key drivers of the redox system. Firstly, the redox-related BPs were obtained by searching the keywords “reactive oxygen species”, “NADPH”, “peroxide”, “superoxide”, “oxidative stress”, and “mitochondrial” in the GO database (https://www.ebi.ac.uk/QuickGO/), while the taxonomy category was restricted to *Homo sapiens*. Relevant BPs were selected in the “Child terms” and “Co-occurring Terms” table. Subsequently, a comprehensive gene list associated with the redox system was extracted by exploring each BPs in “molecular signatures database” (http://www.gsea-msigdb.org/) tab and clicking on “show members”. The genes involved in more than 9 BPs were selected for the subsequent analysis. These selected genes were used in STRING database version 11.5 [21] to identify known protein-protein interactions. The interactions with a combined score >0.5 were chosen for further analysis. Likewise, the TF information associated with these genes was obtained from https://maayanlab.cloud/X2K/.[22]. To evaluate changes in the redox system of covid-19 patients, gene expression profiles of PBMCs from five severe COVID-19 patients and five healthy controls (GSE164805) were analyzed [23]. Pearson correlation coefficients of selected genes from BPs were then calculated based on gene expression data. The final network was constructed based on the multi-level biological data, visualized, and analyzed using Cytoscape v3.8.2 [24]. Top-ranked genes were determined based on the network criteria, including degree and closeness centralities. Moreover, to investigate the distinct and common BPs in which these top-ranked genes are involved, another network of related BPs was constructed by Cytoscape.

### 2.2 Patients

A total of 30 patients with SARS-CoV-2 infection confirmed through real-time reverse-transcriptase–polymerase-chain-reaction (RT-PCR) assays of nasal and pharyngeal swabs were enrolled in this study. The study sample population consisted of NP and whole blood (WB) samples collected in universal transport media and EDTA-coated tubes, respectively, from the clinical laboratories of Taleqani and Firooz Abadi hospitals. Also, WB and NP samples from 30 healthy sex-and age-matched volunteers with a negative PCR result for SARS-CoV-2 infection and no history of COVID-19 were obtained in Taleqani hospital.

The inclusion criteria for participants: (1) not receiving any disease-modifying therapies (DMTs) for COVID-19, (2) age of 18 years or older, (3) SARS-CoV-2 infection was diagnosed using RT-PCR from NP and oropharyngeal (OP) swab, and (4) The patients with COVID-19 and donor controls had not received any antioxidant supplements for at least four weeks before sampling. The participants were informed about the study procedure, and their consent was obtained. The ethics committee approved the study of Shahid Beheshti University of Medical Sciences (SBMU), “IR.SBMU.RIGLD.REC.1399.030.”

### 2.3 PBMC isolation and measuring intracellular metabolites of the redox system in PBMCs using flow cytometry

Ficoll density gradient sedimentation (Lymphodex, Inno-Train, Germany) was used to isolate PBMCs. The production level of intracellular ROS was measured immediately after the isolation of fresh PBMCs by ROS and superoxide detection assay kits (ab139476, USA), as the intracellular ROS & o2-levels change by freezing & thawing. The cells were incubated for 30′ at 37 °C with a permeable green probe (reacts with HO^•^, H2O2, peroxynitrite (ONOO), peroxyradical (ROO^•^), and nitric oxide (NO)) and orange probe (reacts with O_2_^•^^−^). A GSH assay kit (ab112132, USA) was used to determine the level of GSH as an antioxidant molecule. According to the instruction manual, the cells were incubated with thiol green dye at 24 °C for 20 min. BD FACS Calibur™ flow cytometer was used to assess intracellular levels of GSH and ROS/O_2_^•^^−^. Finally, the cells were analyzed based on the difference between the mean fluorescence intensity of ROS/O_2_^•^ − and GSH production in PBMCs of COVID-19 patients and healthy controls by FlowJo software.

### 2.4 RT-PCR

Total RNA was extracted from NP swab and PBMC samples of COVID-19 patients and healthy controls using RNX-plus solution (RN7713C, Sinaclon, Iran) according to the manufacturer's instructions to evaluate gene expression levels of *GP91PHOX* (*CYBB*), NADPH oxidase subunit, and antioxidant enzymes including *CAT, SOD1, SOD2,* and *Nrf2 (NFE2L2)*. NanoDrop (Thermo Fisher) was used to assess RNA concentration and integrity. Subsequently, the total RNA was reverse transcribed into complementary DNA (cDNA) in a reaction primed by a random hexamer, as directed by the manufacturer, using a first-strand cDNA synthesis kit (Parstous, Iran). RT-PCR was performed using 2x SYBR Green qPCR Mix plus (Ampliqone, Denmark) on a Rotor-Gene Q System. Relative expression levels of these genes were normalized by 18s rRNA as a housekeeping gene and calculated by the 2^−*ΔΔ*Ct^ method. The sequences of primers are listed in Table 1.

**Table 1:** Primers for gene expression analysis through real-time PCR.

Gene name	Forward primer (5′→ 3′)	Reverse primer (5′→ 3′)
Gp91 phox	CTGGAAACCCTCCTATGACTTG	GTGATGACCACCTTCTGTTGAG
CAT	TGCTGAATGAGGAACAGAGGAA	CCTCACAGATTTGCCTTCTCC
Nrf-2	CCATTCCTGAGTTACAGTGTCT	CTGTGGAGAGGATGCTGC
SOD1	AGCGAGTTATGGCGACGAAG	CAGCCTGCTGTATTATCTCCA
SOD2	CTCAGGTTGGGGTTGGCT	TGAAGGTAGTAAGCGTGCTCC
18s rRNA	GTAACCCGTTGAACCCCATT	CCATCCAATCGGTAGTAGCG

### 2.5 Measurement of extracellular oxidative stress markers in plasma

WB samples taken from 20 healthy donors and 20 patients were centrifuged at 1000 g for 10 min. Plasma samples were separated and stored at −20 C until analyzed. After that, the plasma levels of total oxidant status (TOS) (NatosTM Total Oxidant Status (TOS) Assay Kit), superoxide dismutase (SOD) activity (Nasdox™–Superoxide Dismutase Assay Kit), and catalase (CAT) (Nactaz™-Catalase Enzyme Activity assay kit- CAT) activity were measured using the commercially available ELISA kits as per the instruction manual.

### 2.6 Discrimination of COVI-19 patients from healthy controls by biomarker panels

Classification analysis was conducted on ELISA results. Logistic regression as a machine learning algorithm was used to build classification models. It was an eligible choice due to being easily trained to work on dichotomous classification problems. A model was constructed for each combination of 1–3 markers (including single CAT, SOD1, and TOS panels, CAT-SOD1, CAT-TOS, and SOD1-TOS panels, and CAT-SOD1-TOS panel), using 5-fold cross-validation in R. The evaluation parameters, such as sensitivity, specificity, and accuracy were calculated by the caret package. pROC package was utilized to plot the receiver operating characteristic (ROC) curves and estimate the area under the ROC curve (AUC), as the threshold independent assessment.

### 2.7 Statistical analysis

Statistical software version 21.0 (SPSS Inc; Chicago, IL, US) was used for all statistical analyses, and data are expressed as means ± standard deviations (SD). Furthermore, REST software (version 2009) was used to analyze gene expression between the studied groups. Results with P-value <0.05 were considered statistically significant.

## 3 Result

### 3.1 A redox system-based gene signature in COVID-19

**Fig. 1:**
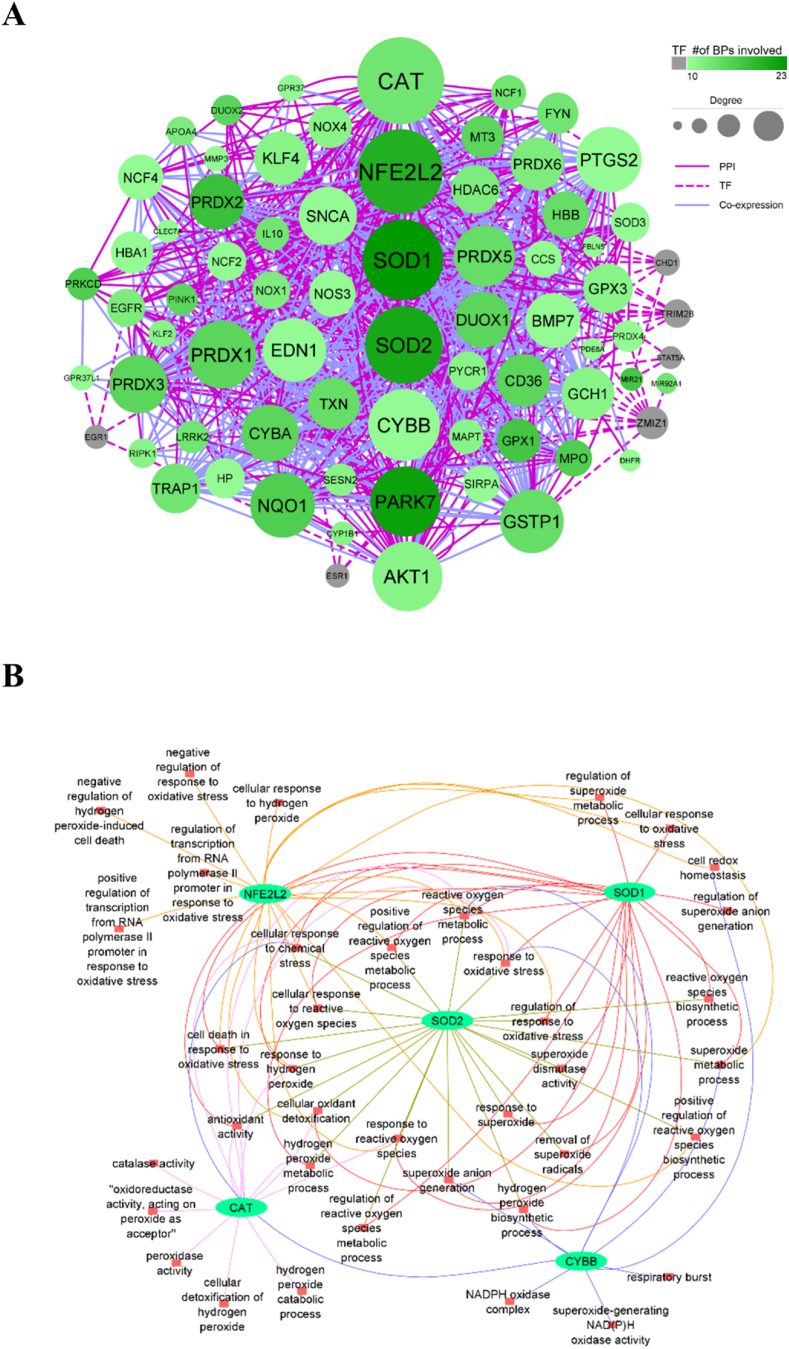
***In-silico* networks. (A)** A multi-level network including three types of edges, PPI, TF, and co-expression. The nodes represent the genes, and the size and color of nodes are associated with the degree and the number of BPs. **(B)** The network indicates BPs in which the top five genes are involved.

The redox system-related BPs were the first biological data exerted in the analysis. 64 BPs were achieved by searching the specific keywords in the GO database for the human organism (Supplementary Table 1). These 64 BPs included 3510 genes and 849 unique genes. The unique genes were involved in 1–23 BPs. We selected 69 unique genes that participated in more than 9 BPs for the subsequent analysis (Supplementary Table 2). The information about PPI and TF related to 69 genes was extracted from corresponding databases. In addition, the co-expression data for unique genes were obtained by Pearson correlation analysis using the gene expression profiles of PBMCs obtained from COVID-19 patients (available inGSE164805). The |correlation coefficient| > 0.8 indicated the significant positive or negative co-expressed genes. The final network was constructed of genes that were connected via PPI/TF/co-expression edges, creating 799 undirected edges between 72 nodes with 3–63° (Fig. 1A).

Although *DHFRP1, H19,* and *MIR675* genes were presented in 10, 16, and 16 redox system-related BPs, respectively, they didn't have any PPI, TF, and co-expression edges with other genes. Consequently, they were excluded from downstream analysis. The network analysis criteria were computed, and the results were presented in Supplementary Table 3. Genes were ranked according to the network criteria, including degree and closeness centralities. The top five genes were *CAT, NFE2L2, SOD1, SOD2, and CYBB (GP91PHOX),* which were involved in 39 BPs (Fig. 1A). The specific and common BPs associated with these high-ranked genes were demonstrated in Fig. 1B (Supplementary Table 4). *SOD1* and *CYBB* which are involved in 23 and 10 BPs had the most and the least contribution to BPs. Response to oxidative stress, reactive oxygen species metabolic process, and cellular response to chemical stress are the common BPs among all five genes (Fig. 1B).

### 3.2 Intracellular redox status: increased expression of oxidative stress molecules in PBMCs of COVID-19 patients

**Fig. 2:**
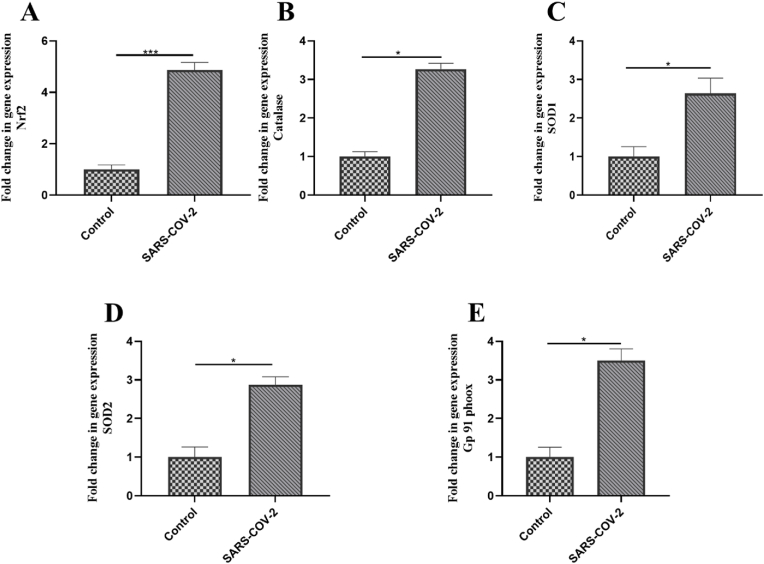
**Expressional changes of oxidant and antioxidant molecules in PBMCs of COVID-19 patients.** The gene expression level of **(A)** gp91-phox, **(B)** Nrf-2, **(C)** CAT, **(D)** SOD1, and **(E)** SOD2 was investigated in PBMCs of COVID-19 patients and healthy controls. An independent sample *t*-test was used to examine the difference across studied groups. *P-value ≤0.05; ***P-value ≤0.001.

To better understand the alteration of oxidative stress-related molecules in the pathogenesis of COVID-19, we validated the top five genes achieved via *in silico* analysis using wet-lab methods. Hence, the gene expression balance of oxidants (NOX-*GP91PHOX*) and antioxidants *(CAT, SOD1 SOD2, and Nrf2)* were investigated in PBMCs. The expression levels of *Nrf2, CAT, SOD1*, and *SOD2* were increased in COVID-19 patients compared to healthy subjects (Fig. 2A–D). likewise, *GP91PHOX* was upregulated in the PBMCs of COVID-19 patients compared to healthy ones. (P-value <0.05) (Fig. 2E). The results have shown that SARS-CoV-2 disturbed the expression of the top five genes related to the redox system in the PBMCs of patients.

### 3.3 Intracellular redox status: increased ROS/superoxide anion and decreased GSH in PBMCs of COVID-19 patients

**Fig. 3:**
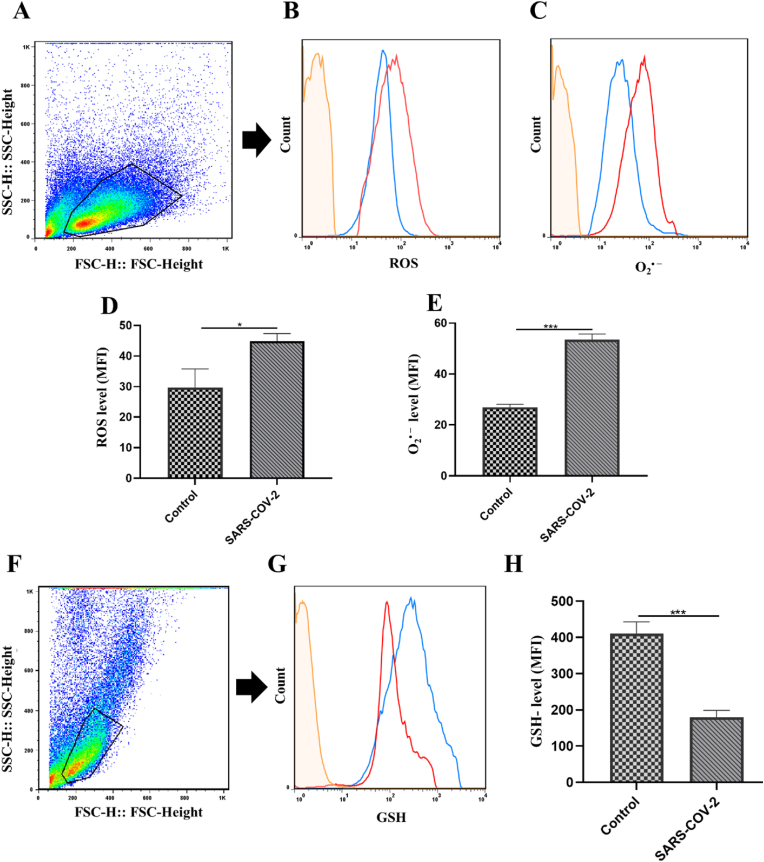
**Changes in ROS/superoxide anion and GSH production in PBMCs of COVID-19 patients. (A)** PBMCs were first gated on a forward vs. side scatter dot plot. **(B)** ROS/**(C)** Superoxide anion production in PBMCs was detected by flow cytometry in unstained (orange line), healthy controls (blue line), and COVID-19 patients (red line). **(D)** ROS and **(E)** Superoxide anion levels in PBMCs isolated from healthy controls and COVID-19 patients. **(F)** PBMCs were first gated on a forward vs. side scatter dot plot. **(G)** GSH production in PBMCs was detected by flow cytometry in unstained (orange line), healthy controls (blue line), and COVID-19 patients (red line). **(H)** GSH intracellular levels in PBMCs isolated from healthy controls and COVID-19 patients. An independent sample *t*-test was used to examine the difference across studied groups. *P-value ≤0.05; ***P-value ≤0.001. MFI: Mean fluorescence intensity.

ROS effects on immunological responses depend on three variables: cell types, intracellular and extracellular ROS. Intracellular ROS, as second messengers, have a role in a variety of cellular activities, including cellular differentiation and the production of pro-inflammatory cytokines. Therefore, to investigate oxidative stress phenomena in PBMCs of COVID-19 patients, we analyzed the intracellular production of ROS/superoxide anion. PBMCs were first gated on a forward vs. side scatter dot plot (Fig. 3A). ROS/superoxide anion producing PBMCs were analyzed on gated PBMCs. The gating strategy to determine ROS/superoxide anion production in gated PBMCs is shown in Fig. 3B and C. Significant higher production of ROS was observed in PBMCs of COVID-19 patients compared to healthy individuals (P-value <0.05) (Fig. 3D). Furthermore, we observed an increased O_2_^•^ − level in PBMCs of COVID-19 patients in comparison to healthy controls. (P-value <0.001) (Fig. 3E). On the other hand, the intracellular GSH, as a major antioxidant in PBMCs, is essential for regulating the activity of redox enzymes and cell cycle progression. Hence, we assessed the intracellular levels of GSH in PBMCs. At first, PBMCs were gated on a forward vs. side scatter dot plot (Fig. 3F). Then, gated PBMCs were analyzed for GSH production (Fig. 3G). Interestingly, a significant decrease in the intracellular level of GSH was observed in PBMCs of COVID-19 patients compared to the control group. (P-value <0.001) (Fig. 3H). Thus, we have found that antioxidant levels were reduced in PBMCs of COVID-19 patients while the oxidant levels were increased, causing oxidative stress in PBMCs of COVID-19 patients.

### 3.4 Extracellular redox status: increased TOS, CAT and SOD activities in plasma of COVID-19 patients

**Fig. 4:**
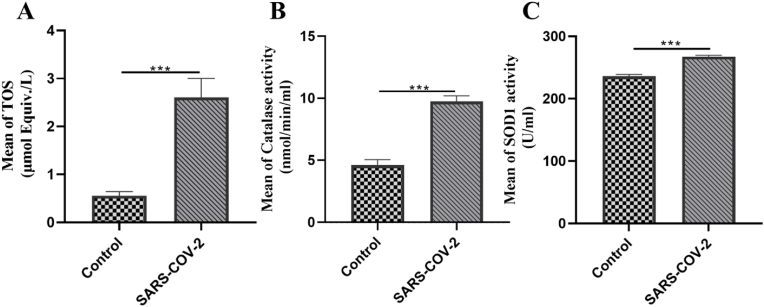
**Changes in the total oxidant status (TOS) and activity of antioxidant enzymes in plasma samples of COVID-19 patients. (A)** The TOS, **(B)** Catalase activity, and **(C)** Superoxide dismutase activity were investigated in the plasma of COVID-19 patients and healthy controls using ELISA. An independent sample *t*-test was used to examine the difference across studied groups. *P-value ≤0.05; ***P-value ≤0.001.

Extracellular redox status is identified as a signaling mechanism that can induce posttranslational changes in redox-sensitive proteins such as enzymes, transcription factors, receptors, adhesion molecules, and membrane signaling proteins, resulting in the regulation of their functions. Moreover, the plasma compartments provide the environment of redox status, which has an important role in circulating immune cells. In addition to PBMCs, we thus evaluated the redox system, including CAT and SOD activities and the TOS in the plasma sample of COVID-19 patients (Fig. 4). The TOS level was significantly higher in COVID-19 patients in contrast to the control group (P-value <0.001) (Fig. 4A). Moreover, according to our findings in Fig. 4B and C, the mean value of CAT and SOD activities was significantly increased in COVID-19 patients compared to the control group (p < 0.001). Despite the antioxidant molecules being upregulated in response to oxidants, they could not alleviate the harmful effects of oxidant molecules.

### 3.5 Redox status in the entry site of SARS-COV-2: decreased gene expression of oxidative stress molecules in NP samples of COVID-19 patients

**Fig. 5:**
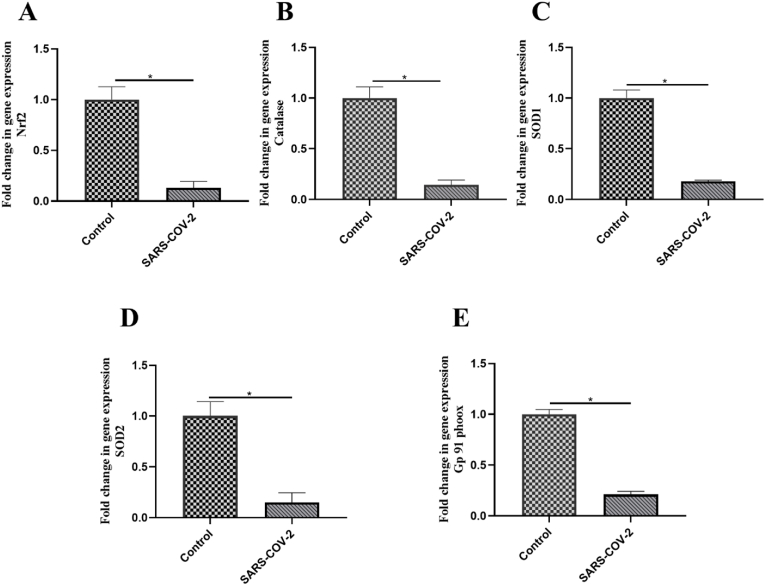
**Expressional changes of oxidant and antioxidant molecules in nasopharyngeal (NP) samples of COVID-19 patients.** The expression level of **(A)** gp91-phox, **(B)** Nrf-2, **(C)** CAT, **(D)** SOD1, and **(E)** SOD2 was investigated in nasopharyngeal (NP) samples of COVID-19 patients and healthy controls using RT-PCR. An independent sample *t*-test was used to examine the difference across studied groups. *P-value ≤0.05; ***P-value ≤0.001.

Since the upper respiratory tract is a known site for SARS-COV-2 entry to host cells, we examined the expression of oxidant (NOX-*GP91PHOX*) and antioxidant genes (*Nrf2, SOD1, CAT, SOD2*) in NP samples to determine whether SARS-COV-2 could disrupt the homeostasis of redox system in the host upper respiratory tract. Remarkably, the results indicated decreased expression of *Nrf2, CAT, SOD1,* and *SOD2* (P-value <0.02) (Fig. 5A–D) as well as *GP91PHOX* (P-value <0.02) (Fig. 5D) in COVID-19 patients compared to controls. Thus, we observed an opposite expression pattern of redox system-related genes in NP samples of COVID-19 patients in contrast to PBMCs and plasma.

### 3.6 Recognition of high-performance diagnostic biomarker panels

According to significant alterations in the TOS and enzyme activities of CAT and SOD in plasma samples of COVID-19 patients, the performance of these markers as discriminators of COVID-19 patients and healthy individuals was investigated by the means of logistic regression as a classifier. Table 2 exhibits the evaluation of models built for each panel.

**Table 2:** Evaluation parameters for the model of each panel.

panel	Sensitivity	Specificity	Accuracy
CAT	0.9	0.9	0.9
SOD1	0.95	0.9	0.925
TOS	0.95	1	0.975
SOD1, TOS	0.9	1	0.95
CAT, SOD1	0.95	0.95	0.95
CAT, TOS	1	1	1
CAT, TOS, SOD1	1	1	1

**Fig. 6:**
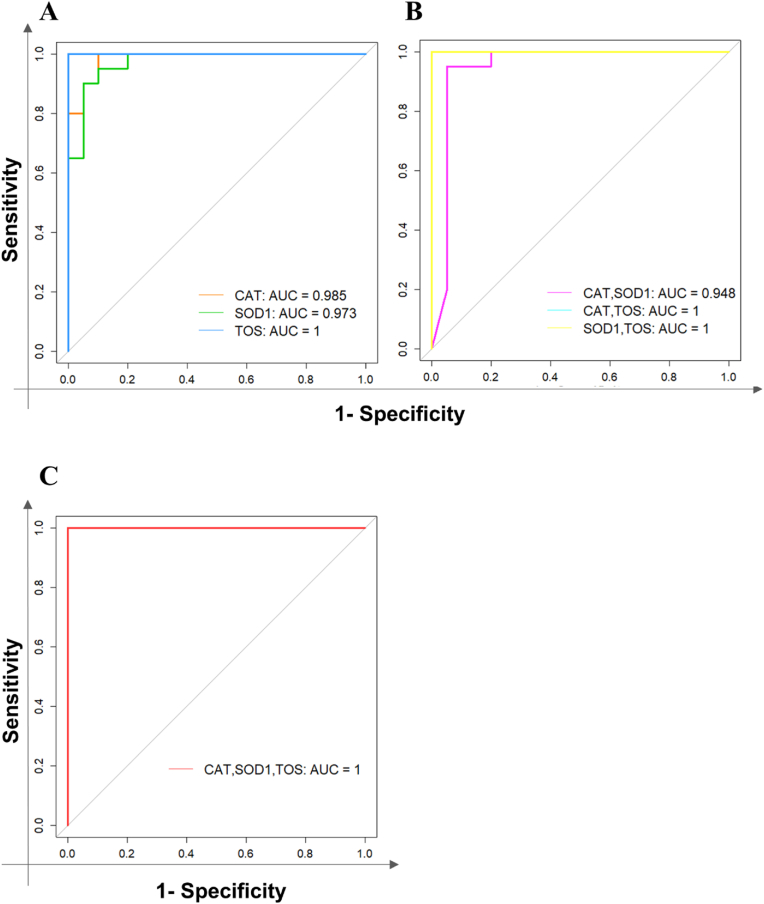
**The ROC curves.** These ROC curves represent the sensitivity vs 1-sprecificity and the AUC of (A) Single biomarker panels, (B) 2-marker panels, and (C) 3-marker panel to discriminate COVID-19 patients from healthy controls.

The reported accuracy for each panel was more than 0.9 that indicating the ability of every panel to accurately detect COVID-19 patients. The sensitivity, specificity, and accuracy rise by increasing the number of panels, reaching the maximum of 1 for all the parameters in CAT, TOS, and CAT, TOS, SOD1 panels. The ROC curves of single, 2-, and 3-marker panels were illustrated in Fig. 6A–C.

## 4 Discussion

SARS-COV-2 infection mainly results in the dysregulation of immune responses and an imbalance in redox homeostasis. Furthermore, it is well known that the redox microenvironment is a critical regulator of immune cell activation and proliferation [25]. Since little is known about extracellular and intracellular redox status in immune cells of COVID-19 patients, we employed integrated multi-level biological data to identify changes in the redox systems of immune cells.

Single-level data analysis alone is not sufficient to achieve a precise understanding of the pathogenesis of a complex disease, necessitating the use of more robust strategies [[26], [27], [28]]. An integrative method that uses multi-level biological data could provide a powerful strategy with more robustness to decipher the mechanistic features of a biological system under pathophysiological conditions and derive new insights into pathogenesis [18]. The BPs employed in this analysis covered various aspects of the redox system, including oxidant and antioxidant systems. Furthermore, most intergenic relationships in BPs known as protein-protein interaction (PPI), are also required for host-pathogen interactions and disease progression [29,30]. Therefore, we also incorporated redox-related PPI and TF information as one of the most vital protein subgroups into the network. To achieve a more comprehensive understanding of redox mechanisms in SARS-COV-2 infection, co-expression data of the redox genes in PBMCs of COVID-19 patients was added as the last data level. Therefore, we found key oxidant and antioxidant genes in the immune cells of COVID-19 patients by network analysis.

Oxidative stress and inflammation are inseparable parts of COVID-19 pathogenesis and progression [31]. Inflammatory reactions induced by SARS-CoV-2 infection impair redox homeostasis and cause high levels of oxidative stress, which in turn maintains the inflammatory state, resulting in a vicious cycle [32]. Oxidative stress can also activate the innate immune response in a chronic manner which is another causing factor of tissue injury in COVID-19 and other infections [33,34]. On the other hand, in adaptive immune responses, oxidative stress can weaken the antiviral activity of CD8^+^ T cells and make CD4^+^ T cells less efficient, leading to low antibody titer [35]. NOX is involved in ROS generation, arterial dysfunction, and thrombosis [36]. Overactivation of NOX has been observed in COVID-19 patients [37]. In this study, we found that the expression of *GP91PHOX*, as a biomarker of NOX activation, was markedly upregulated in PBMCs of COVID-19 patients. To the best of our knowledge for the first time, we measured the intracellular levels of ROS/superoxide anion and GSH as the representatives of oxidant and antioxidant molecules in PBMCs of COVID-19 patients and detected higher oxidant and lower antioxidant levels in patients than in healthy controls. In addition, determining the TOS confirmed a higher concentration of oxidant molecules in the plasma of COVID-19 patients. It seems that these higher levels of oxidant agents can cause the differentiation of pro-inflammatory cells like Th1 cells and induce the production of cytokines like IL-1 β, IL-6, tumor necrosis factor (TNF-α), and IL-8. This inflammatory process can be progressed by stimulating the higher production of oxidative agents in a positive feedback manner [38,39]. Also, other studies have described increasing IFNγ-producing Th1 and a higher level of CD4^+^ T cell activation in COVID-19 patients, leading to worse clinical outcomes [40,41]. Besides, the function of macrophage respiratory burst in response to SARS-COV-2 infection could result in lung tissue damage and epithelial barrier dysfunction via the overproduction of ROS [42,43]. These inflammatory cells and molecules are what cause ''cytokine storm'', lung fibrosis, and declining lung function in COVID-19 [44]. Antioxidant molecules are needed to counteract the effects of oxidant molecules. Previous studies reported that decreased antioxidants, including certain vitamins, enzymes, and trace elements in COVID-19 patients caused oxidative stress and, consequently disease progression [45]. Research on inflammatory lung diseases has indicated that GSH could control the cytokine storm phenomenon by inhibiting NF-kB activation and decreasing ROS production [46]. In this line, we showed that the amount of GSH, which plays an important role in protecting cells from oxidative damage, is significantly diminished in COVID-19 infection. Thus, applying different antioxidants and their analogs could have compensatory effects on disease progression [46,47]. Indeed, SARS-CoV-2 infection disrupts GSH levels and metabolism in the host cell, as well as its role in regulating cellular redox and extracellular thiol homeostasis [48]. Endogenous deficiency of glutathione is regarded to be one of the risk factors that can cause perilous manifestations and even death in COVID-19 patients. For some viral infections like HIV, influenza, and HSV, a low reduced form of glutathione gives the virus a chance to evade the host immune response [49,50]. The studies have reported that the alteration of intracellular GSH in viral diseases leads to an imbalanced Th1/Th2 immune response [51].

Although ROS are frequently produced due to viral infections, antioxidant defenses, such as enzymatic components, are not completely sufficient [52]. SOD and CAT are two important antioxidant enzymes directly engaged in neutralizing ROS and reactive nitrogen species (RNS) [5]. We reported that the expression and enzyme activity of SOD and CAT were incremented in the PBMCs and plasma of COVID-19 patients. Due to the severe inflammatory response and remarkably incremented extracellular and intracellular oxidant metabolites in the blood of COVID-19 patients, it is expected that the level of antioxidant agents increases in response to these conditions; however, it can be inferred that increasing antioxidant molecules are not completely adequate for neutralizing harmful effects of oxidant molecules. Thus, antioxidant-based therapies might manage infectious diseases by inhibiting oxidative stress and consequently improving host immune responses.

Infections with respiratory viruses cause cytopathic effects that are sustained by the viral replication process and the emergence of specific abnormalities in cellular redox [53]. The nasopharyngeal cells are where the SARS-COV-2 enters and replicates. The expression pattern of redox molecules in the upper respiratory tract was completely opposite to what was observed in the whole blood of COVID-19 patients, indicating the function of SARS-COV-2 can be tissue-specific. Studies have documented the associations between influenza virus replication and oxidative stress and have proposed antioxidant agents to suppress virus replication [54]. It has been shown that reducing antioxidant molecules is of great importance for viruses to replicate and cause disease [55]. Furthermore, antioxidant systems prevent the interaction between the cysteine-rich spike glycoproteins of SARS-CoV-2 and its host receptors [48]. Therefore, decreasing antioxidant agents can be an appropriate environment for the entry of SARS-COV-2 into host cells. In this study, we indicated that expression levels of antioxidant molecules were reduced in NP samples of COVID-19 patients. Therefore, the SARS-COV-2 can enter host cells and replicate by preventing the induction of oxidant and antioxidant molecules and the inflammatory response in the upper respiratory tract, which ultimately leads to the escape from immune cells and its survival. Thus, antioxidant treatment may reduce disease outcomes by interfering with viral entry as a critical step in the infection. The reduction of antioxidant agents in NP samples not only shows the difference in the function of the virus in the tissue, the stimulation of cellular responses and the calling of immune cells, but it can also be caused by differences in the resident cells of the target tissue. A recent study showed that granulocytes dominated the nasal immune profile of individuals with acute COVID-19, while other immune cell types such as B cells, dendritic cells (DCs), NK cells, monocytes, CD4^+^ T cells, and CD8^+^ T cells did not statistically alter between individuals with acute infection and healthy controls [56]. This finding that nasal lymphocyte numbers were unchanged is in contrast with immune cell landscape observed in whole blood samples of COVID-19 patients [57].

The conventional diagnostic RT-PCR test for COVID-19, which is based on the detection of viral nucleic acids, suffers from a considerable rate of false-negative results that could be due to the low viral load or poor quality of the sample [58,59]. Consequently, recognizing the host-specific biomarkers as a complementary tool is critical to accurately diagnosing the COVID-19 infection from non-COVID cases. Here we introduced 1 to 3 host-based biomarker panels, including CAT and SOD enzymes and TOS metabolite that could precisely discriminate COVID-19 patients from healthy controls with an accuracy of 0.9–1.

## 5 Conclusion

In conclusion, it seems that SARS-COV-2 deranges the intracellular and extracellular host's redox equilibrium in immune cells, which may lead to dysregulation of host's immune response and a severe form of the disease. On the other hand, SARS-COV-2 can survive and promote the infection by reducing antioxidant/oxidant molecules in the upper respiratory tract and possibly not stimulating the immune system. Therefore, applying antioxidant molecules and their analogs should be considered a treatment option for COVID-19 patients. Moreover, we proposed three oxidative stress markers as hallmarks of SARS-CoV-2 infection that accurately diagnose COVID-19 patients from healthy controls.

## Funding

This study was supported by the 10.13039/501100015693Research Institute for Gastroenterology and Liver Diseases, Shahid Beheshti University of Medical Sciences. The authors also thank the 10.13039/501100003968Iran National Science Foundation (INSF) for partly supporting this research (Grant Number: 99004456).

## Conflicts of interest

M.J.T., H.A.A., M.R.Z., S.M., and A.M have registered a patent related to the diagnostic panels introduced in this article in the Intellectual Property Center of the Islamic Republic of Iran on 09/17/2022 under the number ID140150140003004735. All other authors declare that they have no competing interests.

## Declaration of competing interest

The authors whose names are listed immediately below certify that they have NO affiliations with or involvement in any organization or entity with any financial interest (such as honoraria; educational grants; participation in speakers’ bureaus; membership, employment, consultancies, stock ownership, or other equity interest; and expert testimony or patent-licensing arrangements), or non-financial interest (such as personal or professional relationships, affiliations, knowledge or beliefs) in the subject matter or materials discussed in this manuscript.

## Data Availability

Data will be made available on request.
